# From E. G. B.

**Published:** 1882-12

**Authors:** 


Cincinnati, Nov. 24th, 1882.

Editor Register,

Sir—There appeared in the October number of the Register some criticisms by "Felix" on my report of the American Dental Association meeting of last August. The only point concerning the reporter is "did you ever notice that as funny things occur in reporting as are found in 'the tricks of the types'?" The point he endeavors to make is that the reporter was wrong in making Dr. Watt use a metaphor, viz; "we do know how some medicines act and *we don't know how to* HARNESS *others*" and adds "but Watt is hard to report." (I agree with him in that.) In the November number of the *State Journal* Dr. Watt also denies having used the term. Now, why is it not probable that he did? the reporter was there, pencil in hand. As we all know, Dr. Watt generally speaketh in parables, and when you come to think of it, the metaphor is not at all inapplicable. Besides, the good doctor, some time since, wrote for the Dental Register a most seductive article on horse-shoes, and not long ago was dubbed by somebody 'an old war horse.' Now, if he is an old *war horse* and wears improved shoes to better the grip of his *understanding*, why should he not think well of good *harness*? *

But, joking aside, the error (if it was such) did not in the least affect any statement the doctor made and was not worthy the prominence given it by both editor and correspondent.

I conclude by calling the attention of readers and speakers to the following extract from the editorial columns of the Cincinnati *Commercial* for Nov. 18th, in which there is matter that may also well apply to things and fellows dental:

"It is a common thing for public speakers, whoever say anything worthy of appearing in print, to make complaint of the manner in which they are reported. If they make a mistake to their disadvantage, they endeavor to shield themselves by putting the blame on the reporter. If their long harangues are boiled down into proper strength and flavor to set before an intelligent public, they say their remarks were mutilated and garbled. It is an old dodge—just as editors themselves sometimes excuse their own stupidities by charging them on the "blundering compositor," or "sleepy proof-reader." Mr. Beecher, who for the most part owes his fame as a pulpit orator to newspaper reports of his sermons, had something to say the other day about his having, through a long course of years, been misused by the reporters. The true substance of his complaint was that his many long speeches were not reported in full and in their exact language—as if any newspaper would even publish, or anybody read all Mr. Beecher has had to say since he climbed the pulpit's golden stairs."

